# Temporal Dynamics of Extracellular Matrix Remodeling in Anthracycline-Induced Cardiotoxicity

**DOI:** 10.3390/cells14181471

**Published:** 2025-09-20

**Authors:** Fibi Meshrkey, Somaya Y. Ibrahim, Rushita A. Bagchi, William J. Richardson

**Affiliations:** 1 Ralph E. Martin Department of Chemical Engineering, University of Arkansas, Fayetteville, AR 72701, USA; 2 PharmD Program, College of Pharmacy, University of Arkansas for Medical Sciences, Little Rock, AR 72205, USA; 3 Department of Physiology and Cell Biology, University of Arkansas for Medical Sciences, Little Rock, AR 72205, USA; 4 Division of Cardiovascular Medicine, Department of Internal Medicine, University of Arkansas for Medical Sciences, Little Rock, AR 72205, USA

**Keywords:** anthracycline, doxorubicin, cardiotoxicity, extracellular Matrix, fibrosis

## Abstract

Anthracyclines are widely used chemotherapeutic agents with proven efficacy against a broad range of malignancies, but their clinical utility is limited by a well-documented, dose-dependent cardiotoxicity. While this toxicity has traditionally been attributed to direct cardiomyocyte injury, emerging evidence highlights the pivotal role of cardiac fibroblasts (CFs) in the development and progression of anthracycline-induced cardiotoxicity. This review examines the diverse effects of anthracycline focusing on doxorubicin (DOX) and CFs across the temporal phases of cardiac injury. DOX activates fibroblast-driven extracellular matrix remodeling and promotes fibrosis through enhanced collagen production and the induction of cellular senescence, thereby exacerbating early myocardial inflammation and dysfunction. Clinically, anthracycline cardiotoxicity may present as acute (within days), subacute (within weeks), or chronic progressive forms manifesting either early (within one year) or late (up to decades post-treatment). While early manifestations may be reversible with timely detection and management, late-phase cardiotoxicity is often irreversible, characterized by declining left ventricular ejection fraction and heart failure. A deeper understanding of the molecular and cellular contributions of CFs may uncover novel therapeutic targets to prevent or attenuate anthracycline-related cardiac damage.

## 1. Introduction

Cancer and cardiovascular diseases are the most common cause of death in high income countries [1]. The American Cancer Society estimated the United States would see nearly two million new cancer cases each year [2,3]. A significant proportion of these patients receive systemic chemotherapy as part of their treatment [3]. Although cytotoxic chemotherapy has markedly improved survival across a broad spectrum of malignancies, these regimens frequently cause substantial cardiovascular toxicity [4,5,6,7,8]. Consequently, cardiovascular complications now represent a leading source of long-term morbidity and mortality among cancer survivors. Anthracyclines such as doxorubicin (DOX), daunorubicin, epirubicin, and idarubicin remain fundamental components of therapy for both hematologic malignancies (such as acute leukemias and lymphomas) and several solid tumors (including breast, ovarian, and soft-tissue sarcomas) [5,7,9]. Among these agents, DOX is the most widely used and carries the highest risk for inducing delayed cardiac injury [5,6,7,8,10].

The cardiotoxic effects of anthracyclines are closely related to cumulative dose and manifest across distinct temporal phases [8,9]. Four temporal classifications of anthracycline-induced cardiotoxicity (AIC) are currently recognized: Acute, subacute, early onset chronic, and late-onset chronic [9,11,12,13,14,15]. A study by Cardinale et al. debated that robust data from long term follow up studies validating this classification are lacking, particularly in adult populations [9,11,12]; however, a later retrospective population-based case–control study on patients diagnosed with breast cancer or lymphoma from 1985 through 2010 showed an increased risk of anthracycline-induced cytotoxicity can persist for up to 20 years [16]. The periods between acute and late chronic represents a critical window characterized by evolving structural remodeling and functional impairment, which may be reversible if detected early but can lead to permanent dysfunction if not properly managed.

With a growing population of long-term survivors and the prevalent use of cardiotoxic chemotherapies, there is an urgent need to refine risk-assessment protocols and to implement early preventative or therapeutic strategies. Early investigations focused on direct cardiomyocyte injury and have been extensively reviewed [6,9,10,12,17,18], with some investigation into the role of cardiac fibroblasts (CFs) and the role of extracellular remodeling in AIC [19,20]. However, recent work has identified a crucial role for cardiac CFs and other non-myocyte cell types in the development and progression of anthracycline-induced myocardial damage [6,21,22,23]. This review therefore focuses on the mechanistic involvement of CFs in anthracycline cardiotoxicity, highlighting key signaling pathways that control fibroblast-mediated cardiac remodeling and dysfunction. This review places particular emphasis on the temporal dynamics of these changes, an aspect that has not been systematically addressed in previous work.

## 2. Anthracycline Overview

Anthracyclines were first isolated from a soil-derived Streptomyces species for their antibacterial activity. However, it was not until the early 1960s that their potent antineoplastic properties were discovered through analysis of their chemical characterization [24,25,26]. Anthracyclines are red aromatic type II polyketides featuring a planar aglycone core with tetracyclic ring with adjacent quinone-hydroquinone groups linked covalently linked via O-glycosidic bonds to one or more amino-sugar moieties, with structural diversity arising from modifications to both the aglycone and sugar residue [26,27,28]. Among these, DOX (Adriamycin) and daunorubicin remain the most extensively deployed in clinical oncology, demonstrating broad efficacy against hematologic malignancies such as acute leukemias and lymphomas and solid tumors including breast and ovarian cancer [29,30]. Semi-synthetic analogs [26] like epirubicin, idarubicin, valrubicin and pirarubicin, as well as related synthetic cytotoxic agents such as mitoxantrone [31] and amrubicin [26], have been introduced to expand the therapeutic supply. All these agents share a well-documented, cumulative dose–dependent liability for cardiotoxicity [26].

While the anticarcinogenic effects of anthracyclines have been discussed [9,19,32,33,34,35,36], it remains important to continuously highlight them as the adverse effects on CFs are closely linked to the same molecular mechanisms underlying their anticancer activity. These agents induce cancer cell death through multiple, interconnected pathways, including topoisomerase II inhibition, DNA intercalation, and the generation of reactive oxygen species (Table 1). A key mechanism of their cytotoxicity is the inhibition of topoisomerase II, an enzyme essential for relieving torsional stress during DNA replication resulting in the accumulation of double-stranded DNA breaks. This triggers robust DNA damage responses and ultimately leads to cellular apoptosis [36,37].

Anthracyclines also undergo redox cycling, producing reactive oxygen species (ROS) such as superoxide anions, hydrogen peroxide, and hydroxyl radicals [38,39,40,41]. The reduction in the quinone moiety on the anthracycline molecule initiates this cascade. These ROS cause oxidative damage to nucleic acids, lipids, and proteins [42,43]. While these mechanisms underlie their efficacy against cancer, they also contribute significantly to AIC. The heart is especially vulnerable due to its high mitochondrial content and relatively low expression of antioxidant enzymes [44]. A major contributor to AIC is the high-affinity binding of anthracyclines to cardiolipin, a mitochondrial phospholipid. This disrupts electron transport chain function, impairs ATP synthesis, and triggers the release of cytochrome c, initiating apoptosis in cardiomyocytes [42]. Furthermore, inhibition of cardiac-specific non-proliferative cells topoisomerase IIβ (Top2β) has been shown to impair mitochondrial biogenesis and exacerbate long-term cardiac dysfunction [10,42].

Adverse effects of DOX include both systemic and local toxicities. Systemically, patients frequently experience fatigue, alopecia, nausea, vomiting, and oral sores. Myelosuppression, particularly neutropenia, is nearly universal and contributes to an elevated risk of treatment-related infections. Locally, intravenous administration of DOX can produce progressive tissue injury marked by ulceration and necrosis [29,30]. Cumulative-dose-dependent cardiotoxicity remains the dose-limiting toxicity of DOX. Early changes in myocardial function can evolve into irreversible dilated cardiomyopathy if unrecognized, thus constraining its long-term use [11,12]. Because anthracycline-induced myocardial damage accumulates with successive treatment cycles and is often irreversible once established, it represents a major obstacle to prolonged or repeat anthracycline administration.

AIC refers to the adverse effects of cancer therapies on the heart and circulatory system, encompassing both structural and functional impairments. These range from subclinical changes such as minor alterations in myocardial strain or diastolic function to potentially fatal heart failure. The most widely used diagnostic metric is a decline in LVEF, with cardiotoxicity commonly defined by either a ≥5% drop to below 55% in the presence of heart failure signs or symptoms, or a ≥10% decline to under 55% in asymptomatic patients [9,45,46,47]. Such variability in LVEF-based thresholds underscores the lack of uniform diagnostic standards across clinical trials and practice guidelines.

**Figure 1:**
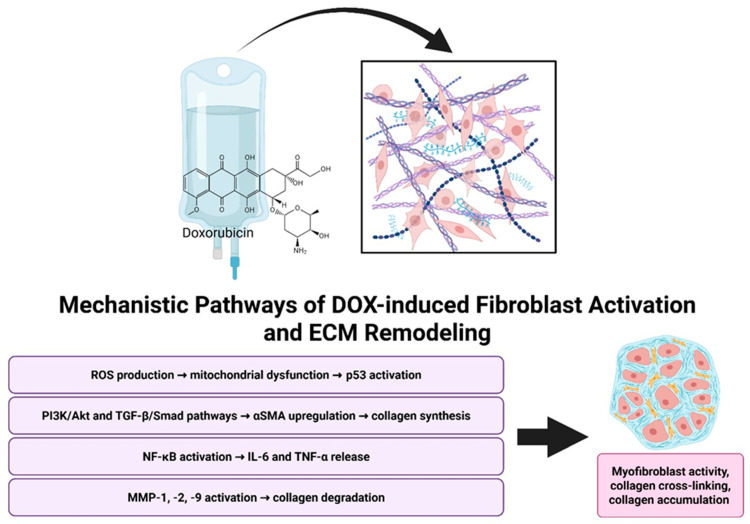
Mechanistic overview of Doxorubicin-induced cardiac remodeling. Doxorubicin promotes fibroblast activation and ECM remodeling through multiple mechanisms, including oxidative stress, pro-fibrotic signaling pathways, inflammatory cytokine release, and MMP-mediated collagen degradation. Together, these processes drive myofibroblast activation, collagen cross-linking, and ECM accumulation. Created in BioRender. Ibrahim, S. (2025) https://BioRender.com/dn6t68x.

AIC arises from multiple, interrelated pathophysiological processes. Central among these are oxidative stress and mitochondrial impairment, which together disrupt cellular homeostasis. Reactive oxygen species inflict damage upon proteins, lipids, and nucleic acids, while compromised mitochondrial bioenergetics precipitates cardiomyocyte apoptosis, necrosis, and senescence (Figure 1) [38,42,48,49]. The cumulative impact of these abnormalities drives acute myocardial injury as well as chronic remodeling and progressive ventricular dysfunction.

## 3. Phenotypic Dynamics

AIC is typically categorized by the latency between drug exposure and the emergence of clinical or subclinical cardiac abnormalities, with acute, subacute, early onset chronic, and late onset chronic phases delineated based on time since the last dose. The precise timing of cardiotoxicity onset is critical for therapeutic strategies, informing timely diagnostic interventions (e.g., echocardiography or biomarker assessment), and optimizing management of cardiovascular adverse events in patients receiving anthracycline chemotherapy [9,11,50]. Cardiotoxic effects of anthracyclines span a clinical spectrum from subclinical myocardial dysfunction to symptomatic heart failure, making early and accurate detection through cardiovascular imaging a cornerstone of cancer care and surveillance. Among available modalities, echocardiography remains the most widely used technique for assessing cardiac function in anthracycline-treated individuals [47,51]. Measurement of baseline LVEF, whether through two- or three-dimensional echocardiography, is well established as a fundamental index of systolic performance and a robust predictor of future heart failure risk in this population [52].

Cardiotoxicity is frequently defined by a serial decline in LVEF, with early AIC often characterized by a ≥10% reduction from baseline values [11,45,47,51]. Patients whose LVEF approaches these thresholds carry a higher risk of progressing to clinical heart failure and may demand initiation of cardioprotective therapies.

AIC results in myocardial ECM expansion due to interstitial fibrosis and inflammation [53]. Cardiovascular magnetic resonance (CMR) imaging allows noninvasive assessment of these changes through quantification of extracellular volume (ECV) fraction. While echocardiographic LVEF remains the clinical monitoring standard [51], its limitations in detecting early tissue changes underscore the value of CMR as a gold standard for evaluating myocardial structure and function, particularly in long-term follow-up.

### 3.1. Acute Phase

Acute AIC typically occurs within hours to two weeks following drug administration and it is relatively rare (<1%) and generally reversible [9,11,17]. Clinically, it may manifest as transient arrhythmias including supraventricular tachycardia, premature ventricular contractions, or atrioventricular conduction delays as well as acute pericarditis or reversible left ventricular dysfunction. In most cases, these effects are self-limiting, resolve upon discontinuation of the drug, and are not dose dependent [29,54,55,56,57]. However, their presence may serve as an early indicator of future cardiotoxicity, emphasizing the importance of thorough cardiovascular monitoring. Acute myocarditis, which can imitate or co-occur with these conditions, is more likely to occur in patients with pre-existing cardiovascular disease or those receiving multi-agent chemotherapy [8,58,59].

Acute cardiotoxicity is characterized by transient reductions in LVEF and a spectrum of arrhythmias, including atrial fibrillation, atrial tachycardia, atrioventricular nodal tachycardia, and premature ventricular contractions [29,54,55,56,57]. Common electrophysiologic abnormalities frequently include sinus tachycardia, repolarization changes, prolonged QT intervals, and reduced QRS voltages [60]. A prospective cohort study of breast cancer patients treated with anthracycline reported that approximately one-third of participants developed acute therapy-related cardiac dysfunction, highlighting the cardiotoxic potential of this regimen [61]. In this study, transthoracic echocardiography was utilized during treatment to detect early functional changes, particularly reductions in LVEF and GLS [61]. CMR demonstrated that early declines in LVEF did not correlate with an increased incidence of symptomatic heart failure during long-term follow-up [61]. These changes usually are associated with transient impairments in myocardial mechanics, including reductions in GLS, indicating diffuse myocardial injury and alterations in left ventricular mass index, suggesting early myocardial remodeling [54,62].

### 3.2. Subacute Phase

Subacute AIC refers to an intermediate rare phase of myocardial injury that typically develops within weeks to a few months after treatment [15,50,63]. It serves as a transitional stage between the transient acute effects and the possible progression to early onset chronic cardiomyopathy. Clinically, this phase is often asymptomatic or represented as indistinct chest pain [50], characterized by subclinical reductions in LVEF detectable only through sensitive imaging modalities such as global longitudinal strain (GLS) or via biomarker surveillance [11].

In a reported case, a 25-year-old male developed subacute cardiotoxicity 17 days after receiving anthracycline therapy and presented with transient left ventricular (LV) wall thickening on echocardiography that resolved within one month [50]. Despite preserved systolic function, the patient exhibited significantly reduced early diastolic velocities, suggesting isolated diastolic dysfunction as a subclinical manifestation of myocardial injury. Although the cardiac abnormalities were resolved without progressing to chronic dysfunction, this case emphasizes the potential utility of diastolic functional changes as an early indicator of anthracycline-related myocardial stress [50].

If myocardial dysfunction in this phase left unrecognized or unmanaged, persistent myocardial injury can progress into early onset chronic cardiotoxicity most commonly presenting as dilated cardiomyopathy within the first-year post–anthracycline exposure [17].

### 3.3. Early Onset Chronic Phase

Early onset chronic anthracycline-induced cardiotoxicity is the most observed clinical presentation, typically manifesting within the first year after treatment completion [9,17]. Cardinale et al. have suggested that the distinction between early and late-onset chronic cardiotoxicity may be misleading, as many cases historically labeled as chronic could have been detected earlier with modern tools and possibly reversed with timely cardioprotective therapy [11,51]. While this perspective emphasizes the value of early detection and intervention, it is equally important not to overlook the distinct subacute phase and early chronic phase. Recognition of these subclinical windows is critical, as they represent a pathophysiologically active yet clinically silent phases during which timely interventions may prevent permanent cardiac damage and improve long-term outcomes.

This phase is clinically characterized by asymptomatic reductions in LVEF within the first-year post-treatment, and accounts for most cases in adult cohorts [59]. Despite its utility, LVEF lacks sensitivity for detecting early myocardial injury, prompting greater reliance on more sensitive imaging biomarkers. Speckle-tracking echocardiography (STE), particularly GLS, has emerged as a powerful tool for detecting subclinical myocardial dysfunction prior to LVEF decline [64,65]. A GLS value below 16% or a relative reduction >15% from baseline has been shown to signal elevated cardiotoxicity risk and may justify initiation of cardioprotective medications [11,47,51]. Diastolic dysfunction is increasingly recognized as an early marker of AIC, often preceding detectable systolic impairment [55,66,67,68].

### 3.4. Late Onset Chronic Phase

Chronic AIC is defined by its delayed onset, often developing years to decades after completion of chemotherapy [9,11]. This prolonged latency is usually accompanied by irreversible structural and functional cardiac alterations, such as myocardial fibrosis and ventricular remodeling, and is associated with poor long-term outcomes, including increased cardiovascular mortality [51]. The risk of chronic cardiotoxicity displays a strong dose-dependent relationship, with heart failure incidence rising from a maximum of ~5% at cumulative anthracycline doses below 400 mg/m^2^ to over 18% at doses of 700 mg/m^2^ [8,12,69]. A sign of chronic toxicity is the progressive decline in left ventricular systolic function, which may end in dilated cardiomyopathy and symptomatic heart failure [51]. In long-term cancer survivors, subclinical indicators including impaired diastolic relaxation on echocardiography, reduced myocardial strain, and modest elevations in biomarkers are frequently detectable before overt symptoms arise [11,51].

Reductions in CMR-derived LVEF by >10%, particularly when falling below 53%, are considered indicative of therapy-related cardiac dysfunction [70]. Parametric techniques such as native T1 mapping, T2 mapping, and the extracellular volume (ECV) quantification offer unique insights into myocardial edema, diffuse fibrosis, and interstitial expansion thereby enhancing diagnostic precision and prognostic stratification [9,71]. In chronic AIC, CMR imaging has demonstrated that increased ECV fractions, indicative of diffuse interstitial fibrosis, correlate with impaired systolic and diastolic function, reduced exercise capacity, and increased long-term mortality even up to twenty years after anthracycline exposure in long-term survivors of childhood cancer [51,61,72]. These findings emphasize the need for cardiovascular surveillance, even in asymptomatic individuals, to enable early detection and intervention.

## 4. Molecular Dynamics

Anthracyclines remain cornerstone chemotherapeutic agents for a range of malignancies, but carry a well-characterized, dose-dependent risk of cardiotoxicity that may end in irreversible cardiac dysfunction and heart failure [7,20,23,32]. AIC activates proteolytic and inflammatory pathways that drive extracellular matrix (ECM) degradation, deposition, and temporal morphological changes in the myocardium [73]. Histological studies using Picrosirius red staining under polarized light have demonstrated that DOX increases interstitial collagen deposition, an effect that is largely attenuated by pharmacologic inhibition of matrix metalloproteinases with agents such as doxycycline [23,73,74].

Conventional cardiac imaging methods, while essential for patient surveillance and follow-up, often detect cardiotoxicity only after substantial and potentially irreversible myocardial damage has already occurred, driving interest in molecular indicators for earlier, subclinical detection. Circulating biomarkers can rise within days of anthracycline exposure before detectable changes in echocardiographic parameters are apparent emphasizing their sensitivity to early myocardial injury [75]. Some of these biomarkers specifically reflect fibroblast activity, while others indicate broader myocardial injury but still participate in fibroblast-mediated fibrotic cascades. Among these, matrix metalloproteinases (MMP-2, MMP-9) and their inhibitors (TIMP-1, TIMP-2) reflect fibroblast-driven extracellular matrix turnover and remodeling [73,76,77,78].

### 4.1. Acute Phase

In animal models of acute anthracycline injury, myocardial histopathology is marked by dense interstitial inflammatory infiltrate composed predominantly of neutrophils, lymphocytes, and macrophages [72,79,80]. Clinical evidence aligns with these findings.

In vitro, primary CFs exposed to DOX demonstrated increased α-smooth muscle actin (α-SMA) expression and enhanced collagen synthesis, signifying early myofibroblast differentiation and enhanced ECM production [67]. Animal models have provided valuable insights into the temporal evolution of DOX-induced myocardial injury. In murine studies, the acute phase is characterized by prominent intracytoplasmic vacuolation of cardiomyocytes, particularly within the ventricles and interventricular septum, along with occasional myofiber atrophy indicating early structural disruption [72]. Myocardial cells after acute exposure showed swelling, sarcoplasmic core dilation, myofibril loss, cytoplasmic vacuolization, and enlarged nuclei with prominent nucleoli [81].

Circulating biomarkers capture the extent of this injury. High-sensitivity troponin is the earliest and most reliable indicator of acute myocyte necrosis; even modest troponin elevations identify patients at heightened risk for subsequent chronic cardiotoxicity [82]. Cardiac troponin I (cTnI) and troponin T (cTnT) are the most sensitive circulating markers of acute cardiomyocyte injury in patients receiving anthracycline chemotherapy. cTnI levels begin to rise within 24 h of each anthracycline dose and demonstrate a cumulative increase with successive cycles, reflecting ongoing subclinical myocardial injury [72,82,83]. Persistent elevation of cTnI or cTnT during or immediately after completion of therapy identifies individuals at significantly higher risk for subsequent declines in LVEF and overt cardiotoxicity [56,83,84]. These troponin elevations are often transient and may normalize if therapy is held, but their presence should prompt consideration of cardioprotective interventions (e.g., ACE inhibitors, β-blockers) [85].

### 4.2. Subacute Phase

Owing to the markedly accelerated course of anthracycline cardiotoxicity in animals, especially mice, histological reports of subacute injury are limited and often indistinguishable from features of acute or early onset chronic injury, a notable case report [50] detailed subacute anthracycline cardiotoxicity demonstrated that an endomyocardial biopsy performed 17 days after consolidation chemotherapy revealed marked interstitial myocardial edema without evidence of inflammatory cell infiltrates or myocyte necrosis and only minimal interstitial fibrosis suggestive of a potentially reversible stage of injury. Cellular infiltration may be a secondary response, occurring only after overt myocyte injury has taken place [50]. In a rabbit model, Bristow et al. found that subacute injury histology resembled acute phase features with predominant neutrophilic infiltration, occasional eosinophils, focal areas of myocyte degeneration, and interstitial edema [15]. On the biomarker level, case reports describe elevations in troponin and C-reactive protein (CRP) during this interval, indicating ongoing myocardial injury without full progression to chronic damage [50,86].

### 4.3. Early Onset Chronic Phase

In contrast to the acute phase, the early onset chronic stage of DOX exposure (≥12 weeks) is characterized by more extensive myocardial remodeling. Zeiss et al. reported features including myofiber disorganization, marked variability in myofiber diameter, persistent vacuolation in some animals, and the development of fine interstitial “chicken-wire” fibrosis indicating progressive structural damage [72]. Typically, this phase is clinically silent but detectable by rising natriuretic peptides (BNP/NT-proBNP) and inflammatory markers (e.g., high-sensitivity C-reactive protein, GDF-15, sST2, MPO).

B-type natriuretic peptide (BNP) and its N-terminal fragment, NT-proBNP, are released in response to increased ventricular wall stress and can rise within days to weeks after initiation of anthracycline therapy, often before echocardiographic evidence of dysfunction appears [87]. Serial measurements of NT-proBNP during chemotherapy have demonstrated that persistently elevated or progressively increasing levels predict both early and late left ventricular dysfunction [87,88]; for example, a rise of ≥36% from baseline in NT-proBNP during treatment correlates with a significantly greater likelihood of LVEF decline at 3, 6, and 12 months post-therapy [88]. In anthracycline-treated cohorts, elevated NT-proBNP immediately post-cycle has been associated with subclinical left ventricular impairment, and patients with persistently high NT-proBNP levels exhibit worse ventricular remodeling and a higher incidence of heart failure on follow-up [87,88]. Early recognition during this window allows timely initiation of cardioprotective therapy to prevent irreversible injury [75,88].

Inflammatory and oxidative stress biomarkers have so far been characterized primarily in preclinical models and few cohort studies [75,86,89]. They also have been previously discussed in the literature [9,90,91,92,93]. However, their clinical relevance remains to be confirmed through well-designed cohort and clinical studies.

### 4.4. Late Onset Chronic Phase

Prolonged anthracycline exposure leads to cumulative left ventricular dysfunction characterized by maladaptive remodeling. Over months to years, cardiomyocyte hypertrophy, mitochondrial impairment, and disorganization of the myocardial ECM give rise to chamber dilation and heart failure symptoms with reduced ejection fraction [12,35,47,53,94]. Histologically, this chronic stage is defined by diffuse interstitial fibrosis alongside an imbalance in matrix metalloproteinase (MMP-2/MMP-9) activity relative to their tissue inhibitors (TIMP-1, TIMP-2), promoting excessive collagen accumulation [4,20,78]. Upstream mediators such as TNF-α, IL-6, and ROS further amplify MMP expression and activation, integrating inflammatory and redox signals into the fibrotic remodeling characteristic of AIC [20,78].

The late-onset cardiotoxicity which emerges years after treatment completion is characterized by elevated novel fibrosis-related biomarkers such as PICP and galectin-3 [9,75,95]. Circulating procollagen peptides specifically the carboxy-terminal propeptide of procollagen type I (PICP) and the amino-terminal propeptide of procollagen type III (PIIINP) serve as indicators of myocardial collagen synthesis and interstitial fibrosis [96,97]. In women undergoing anthracycline-based breast cancer chemotherapy, early increases in PICP at three months post-treatment were significantly associated with subclinical reductions in LVEF and predicted development of overt cardiotoxicity at one year, whereas NT-proBNP alone showed less prognostic value [95]. PIIINP rise typically occurs after established ventricular dysfunction, limiting its utility for early detection [96].

## 5. Fibroblasts and Extracellular Dynamics

### 5.1. Cardiac Fibroblasts

In the healthy myocardium, CFs represent the predominant non-myocyte cell type alongside endothelial cells, vascular smooth muscle cells, and transient leukocytes and are indispensable for maintaining ECM integrity and providing mechanical support [21,98,99]. Although early investigations of DOX cardiotoxicity concentrated on direct cardiomyocyte injury as the main pathogenesis [6,37], emerging evidence has expanded the view by implicating the direct involvement of CFs and non-myocytes cellular constituents in AIC affecting fibroblast function, disrupting ECM homeostasis, provoking inflammatory signaling, driving maladaptive remodeling and in the initiation and propagation of chronic myocardial damage [21,23,100]. Collectively, these findings support the notion that fibroblast-driven mechanisms may be central to both the development and progression of long-term anthracycline cardiotoxicity.

The mechanism by which anthracyclines, particularly DOX, adversely affect CFs closely parallels their antitumor activity. Cardiac fibroblasts are highly susceptible to doxorubicin-induced oxidative stress, primarily mediated by ROS generated through mitochondrial complex I [101]. The produced lipid peroxidation, protein oxidation, and activation of stress-responsive kinases such as p38 MAPK drive apoptosis, cellular senescence, and phenotypic reprogramming toward a pro-inflammatory and profibrotic state [21,67,94].

Alteration in mitochondrial metabolism including cardiolipin remodeling have been proposed not only as a potential mechanism of AIC but also underlying sex-related differences [102]. The influence of sex and age on AIC remains incompletely characterized. Nevertheless, accumulating evidence from experimental and retrospective cohort studies indicates that these variables are major modifiers of susceptibility and should be explicitly integrated into preclinical study design, patient risk stratification, and the development of cardioprotective strategies [102,103,104,105,106]. Most experimental data in pediatric oncology suggest a higher incidence of AIC among females compared with males, whereas limited adult data raise the possibility of a sex-related protective effect in women [105].

Sex-related disparities extend to metabolic and structural outcomes under cumulative anthracycline exposure. Male rodents exhibit greater weight loss, reduced survival, pronounced myocardial dysfunction, impaired energy signaling, decreased mitochondrial biogenesis, lower cardiolipin content, diminished mitochondrial DNA, and altered mitochondrial respiration, accompanied by a stronger upregulation of myocardial stress biomarkers compared with females [104].

ECM remodeling also appears to contribute to these differences; although fibrosis surrounding cardiac muscle cells is comparable between sexes, total and reactive fibrosis are significantly greater in males, suggesting a sex-dependent propensity for adverse fibrotic remodeling [104]. These findings underscore the need for standardized diagnostic criteria and prospective studies specifically designed to study sex-based differences in AIC.

Age further modulates anthracycline toxicity. Preclinical studies consistently demonstrate a dose-dependent escalation of cardiotoxicity with advancing age. In an animal model, older rats (24 months) displayed markedly greater vulnerability to anthracycline-induced myocardial injury than younger animals (6 months) exposed to comparable doses [103].

Recent sequential studies by Ishikawa group [22] have demonstrated that sublethal concentrations of DOX induce trans-differentiation of human cardiac fibroblasts (HCFs) into myofibroblasts, characterized by increased expression of α-SMA, matrix metalloproteinase-1 (MMP-1), interleukin-6 (IL-6), and collagen. These changes are mediated via activation of the PI3K/AKT and TGF-β/Smad signaling pathways [22]. In that experiment comparing HCFs and normal human dermal fibroblasts (NHDFs), the α-SMA-mediated trans-differentiation into myofibroblasts is specific to HCFs and is absent in NHDFs except after direct TGF-β stimulation, indicating a greater susceptibility of HCFs to doxorubicin-induced fibrotic remodeling [22]. Further supporting the role of CFs in DOX-induced cardiotoxicity, these studies show that systemic administration of DOX leads to activation of fibrotic signaling pathways in CFs, including upregulation of Toll-like receptor 9, interleukin-1 (IL-1), and fibrotic markers such as galectin-3, α-SMA, and tissue inhibitor of metalloproteinases-1 (TIMP1) [21,22,94]. Skaggs et al. used proteomic profiling to show that DOX-treated primary CFs exhibit impaired ECM maintenance, marked by downregulation of ECM-regulatory genes including Col4a1, Col4a2, Col5a1, Mmp11, Mmp14, and TGFβ, indicating disruption of basement membrane integrity, matrix organization and augmented myofibroblast differentiation [23].

### 5.2. Effects of Anthracylcine on Fibroblast Signaling

Transforming growth factor-beta 1 (TGF-β1) is a central mediator in the conversion of fibroblasts to myofibroblasts and drives collagen synthesis within the myocardium. In models of anthracycline-induced cardiomyopathy, TGF-β1 and its downstream effector, SMAD3, are upregulated two- to four-fold at both early and late time points in the course of the disease [20]. In a meta-analysis of AIC in animal models, Leerink et al. reported that connective tissue growth factor (CTGF), a canonical TGF-β1 target, peaks shortly after anthracycline exposure and remains elevated during long-term follow-up [20]. Additionally, transcripts encoding fibroblast activation markers such as osteonectin and tenascin-C are upregulated in large-animal studies [20,98,107]. These findings, along with persistent upregulation of collagen types I and III and progressive interstitial fibrosis, underscore the enduring nature of pro-fibrotic signaling in anthracycline-induced cardiomyopathy [108]. DOX elicits an early inflammatory cytokine response in CFs, followed by late-phase collagen production, thereby driving the sequential stages of anthracycline-induced myocardial remodeling [67]. DOX prompts HCFs to secrete pro-inflammatory cytokines and pro-fibrotic growth factors. Specifically, IL-6 and TGF-β mRNA rises in a time-dependent manner over the first 3–6 h of DOX stimulation [22]. DOX stimulates inflammatory and fibrotic pathways in HCFs via both PI3K/AKT and TGF-β/Smad signaling cascades, as evidenced by time-dependent phosphorylation of Smad2 and AKT following DOX exposure. Moreover, HCFs demonstrate a progressive increase in Col1a1 transcript levels across 72 h of DOX treatment [22]. Inhibition of TGF-β/Smad signaling attenuates DOX-induced IL-6 mRNA expression and α-SMA protein levels, while blockade of PI3K selectively suppresses MMP-1 protein upregulation in HCFs [22].

AKT, a serine/threonine kinase, plays a pivotal role in regulating cardiomyocyte survival, growth and function. Activation of the PI3K/AKT pathway is essential for normal cardiac growth, as well as for the heart’s adaptive responses to stress [109,110,111]. Transient activation of AKT has been shown to confer cardioprotection by inhibiting apoptosis and inducing adaptive functional hypertrophy characterized by preserved contractile function [112,113], While prolonged AKT signaling results in extensive cardiac hypertrophy, interstitial fibrosis, and impaired contractile function [112]. These pathological changes are associated with a mismatch between cardiomyocyte growth and angiogenesis, leading to reduced capillary density and subsequent cardiac dysfunction. In experimental models of anthracycline-induced cardiomyopathy, a decrease in phosphorylated AKT levels has been observed, indicating compromised pro-survival signaling. This reduction in AKT activity undermines the heart’s endogenous protective mechanisms, exacerbating ECM perturbations and contributing to the progression of cardiac injury [20,114].

In addition to TGFβ and AKT signaling changes, DOX also induces the generation of ROS, leading to oxidative stress and mitochondrial dysfunction [38]. This oxidative stress activates p53, a tumor suppressor protein that regulates cell cycle arrest and apoptosis [115,116]. In cardiac fibroblasts, p53 activation contributes to cellular senescence and the senescence-associated secretory phenotype (SASP), which includes the secretion of pro-inflammatory cytokines and pro-fibrotic factors [37,117]. These processes further exacerbate myocardial remodeling and fibrosis [37,118]. Using primary cardiac fibroblasts isolated from murine hearts, Mancilla and colleagues demonstrated that doxorubicin markedly suppresses fibroblasts expansion, reducing cell confluence by nearly 50% relative to untreated controls within 72 h in both wild type and P53 -/- cells [118]. Time-course analysis indicated that by 36 h the loss of confluence reflected predominantly cell death rather than reduced proliferation. In addition, doxorubicin reduced migration in wild-type fibroblasts, whereas genetic deletion of p53 restored migratory capacity. Wild-type cells exhibited a significant shift in cell-cycle distribution, consistent with arrest at the p53-dependent G1/S checkpoint, while p53 -/- fibroblasts did not display the same arrest, indicating that their decreased proliferation arises via alternative mechanisms. Beyond these functional effects, the study revealed a profound disruption of mitochondrial quality control. Doxorubicin markedly increased p53 protein levels and promoted its cytosolic accumulation increasing P53/parkin interaction. This prevented Parkin from localizing to damaged mitochondria, effectively blocking mitophagy, leading to mitochondrial dysfunction and accumulation of reactive oxygen species [118]. In addition, previous in vitro studies have demonstrated that doxorubicin markedly reduces cardiac fibroblast numbers predominantly by inhibiting proliferation rather than inducing cell death. Cell counts at 24, 48 and 96 h post-exposure show a pronounced reduction in proliferation, accompanied by significant downregulation of cyclin D1, consistent with doxorubicin-induced G_1_/S cell-cycle arrest in fibroblasts [119].

### 5.3. Effects of Anthracycline on ECM Remodeling

The cardiac ECM is a dynamic and intricate network composed primarily of fibrillar collagens (types I and III), along with proteoglycans, glycoproteins, and glycosaminoglycans, which together provide structural and biochemical support to the myocardium [120]. In addition to acting as a fibrous scaffold, the ECM serves essential mechanobiological roles, facilitating force transmission, limiting myocardial overstretch, and preserving the structural integrity of cardiomyocytes and the intramyocardial vasculature [98,99,120]. Cardiac fibroblasts are central regulators of ECM homeostasis, capable of sensing biomechanical and biochemical cues and modulating the synthesis and degradation of ECM components accordingly [98,99]. However, this regulatory system becomes profoundly disrupted upon exposure to anthracyclines such as DOX, which induces dose-dependent cardiotoxicity characterized by cardiomyocyte loss, interstitial fibrosis, and exaggerated inflammatory signaling, ultimately leading to maladaptive cardiac remodeling [6,22,67,94,121].

DOX-induced oxidative stress and inflammatory signaling activate matrix metalloproteinases, enzymes that degrade ECM components, promote myofibroblast differentiation, and drive excessive collagen deposition (Figure 1) [22,67,94,118]. Disruption of ECM integrity, whether through excessive proteolysis or unrestrained matrix accumulation, destabilizes the myocardial architecture [122]. Excess collagen leads to interstitial fibrosis and vascular encasement, while matrix degradation weakens the collagen scaffold, impairing systolic and diastolic function and disrupting electrical synchrony [123]. Notably, anthracycline-induced ECM remodeling is temporally heterogeneous. Acute DOX exposure is associated with transient fibroblast activation and matrix degradation, whereas chronic exposure leads to persistent myofibroblast differentiation, pathological collagen accumulation, and irreversible fibrotic remodeling [22,23,53,94,95]. In vitro, DOX elicits a temporal response in HCFs; within 3–6 h, there is an increase in IL-6, IL-1β, and TGF-β mRNA expression, followed by upregulation of α-SMA mRNA at 24 h and α-SMA protein accumulation by 48 h culminating in collagen deposition [22,94].

Matrix metalloproteinases (MMPs) constitute a family of zinc-dependent endopeptidases that play pivotal roles in the degradation and remodeling of the ECM. These enzymes are integral to both physiological processes such as embryonic development, angiogenesis, and wound healing and pathological conditions, including tumor invasion, atherosclerosis, myocardial infarction, and heart failure [77]. In the context of cardiac tissue, several MMPs have been identified, including MMP-1, MMP-2, MMP-7, MMP-9, and MMP-14 [77,124]. Each of these enzymes exhibits distinct substrate specificities and regulatory mechanisms, contributing variably to ECM turnover and cardiac remodeling. While some MMPs are non-specific to the heart, MMP-1, 2 and 9 are directly involved with AIC [20,22,23,77,78].

MMP-1, also known as interstitial collagenase, is primarily responsible for initiating the degradation of fibrillar collagens, particularly types I and III; the principal structural proteins of the cardiac ECM [124]. Its overexpression has been implicated in pathological cardiac remodeling as it contributes to excessive ECM degradation, undermining myocardial structural integrity and promoting ventricular dilation [76]. Experimental models have shown that upregulation of MMP-1 is associated with the development of cardiac hypertrophy and progression to decompensated heart failure [122,125,126]. DOX exposure increased MMP-1 expression through the activation of the PI3K/Akt signaling pathway [22]. This abnormal upregulation of MMP-1 in HCFs contributes to ECM destabilization, fibroblast activation, and ultimately the fibrotic and functional decline observed in AIC [22].

MMP-2 and 9, also known as gelatinases, are fundamental to cardiac ECM turnover and remodeling and play dual roles (protective or pathogenic) depending on their activation timing and cellular localization [77,124]. MMP-2 is constitutively expressed in cardiomyocytes and localizes to subcellular compartments including sarcomeres, mitochondria, nuclei, and cytoskeleton, where it contributes to both normal cell signaling and structural integrity [77,78]. Anthracycline exposure, particularly to DOX, induces oxidative stress that activates latent MMP-2 and MMP-9, increasing their enzymatic activity and driving pathologic remodeling [77,94]. DOX also triggers de novo synthesis of MMP-2 and upregulates a pathological splice variant known as N-terminal truncated MMP-2 (NTT-MMP-2), which lacks the mitochondrial targeting sequence [127,128]. This variant promotes mitochondrial dysfunction, activates nuclear factor κB (NF-κB), and increases the expression of interleukin-6 (IL-6), exacerbating myocardial inflammation and injury [67]. Once activated, MMP-2 and MMP-9 degrade key contractile proteins such as troponin I and myosin light chain-1, leading to impaired sarcomeric integrity and reduced cardiomyocyte contractility [129]. Animal models have demonstrated that even a single dose of DOX significantly reduces LVEF and fractional shortening, with early diastolic dysfunction evident alongside increased myocardial MMP activity [67].

### 5.4. Acute Phase

Acute DOX exposure rapidly disrupts ECM homeostasis via a combination of transcriptional regulation, oxidative stress, and inflammatory signaling in CFs. A key driver of this phenotype is DOX-mediated redox cycling, which generates excessive reactive oxygen species (ROS), leading to mitochondrial dysfunction, lipid peroxidation, and DNA damage. These ROS also activate p38 MAPK and JNK/NAD(P)H oxidase pathways, resulting in the early upregulation of MMP-2 and MMP-9 and subsequent ECM breakdown [42,126,130,131] (Table 2). Within four weeks after a single therapeutic dose of DOX, myocardial cytoskeletal proteins including tubulin, fibronectin, and myosin light-chain kinase are markedly reduced, although total interstitial collagen volume remains unchanged. This suggests that early subcellular remodeling proceeds overt fibrosis [132]. Concurrently, DOX elicits ROCK1-dependent actin cytoskeletal reorganization in CFs, leading to cell shrinkage, detachment, and apoptosis [133].

Experimental studies showed that DOX downregulates cellular communication network factor 2 (CCN2) in both NIH3T3 embryonic fibroblasts and primary CFs indicating a potential for early antifibrotic activity [100]. Consistent with this observation, proteomic and transcriptomic profiling of primary murine CFs treated with 1 μM DOX for 24 h demonstrated acute downregulation of extracellular matrix (ECM)-related genes, indicating compromised basement membrane integrity and matrix cohesion [23]. These transcriptional changes coincide with reduced collagen deposition and gross ECM destabilization in vivo. In parallel, increased activities of MMP-1, -2, and -9 contribute to myocardial fibrosis, collagen disarray, and cytoplasmic vacuolization [4,22,76,131].

In an in vitro study, CFs exhibited a shift toward a myofibroblast phenotype within 24 h of DOX exposure, characterized by early induction of α-SMA and subsequent collagen synthesis, driven by autocrine and paracrine TGF-β signaling and SMAD2 phosphorylation [67]. This myofibroblast activation spreads a pro-fibrotic loop, while ECM stiffening alters mechano-electrical coupling, potentially promoting arrhythmogenesis [134,135,136]. Finally, NF-κB signaling is triggered early, raising TNF-α and IL-6 levels a response that can be attenuated by FGF21 by reducing NF-κB p65 nuclear translocation [137].

### 5.5. Subacute Phase

Although infrequently characterized, the subacute phase marks a transitional period where CFs begin to exhibit a pro-inflammatory and proteolytic phenotype in response to anthracycline exposure. During this phase, increased vascular permeability and interstitial edema arise, driven by a Toll-like receptor 2 (TLR2)-mediated inflammatory response. DOX activates TLR2 signaling in endothelial cells, upregulating pro-inflammatory cytokines such as TNF-α and IL-6. [50,138]. This inflammatory amplification, while less extensively studied than in the acute or chronic phases, likely contributes to ongoing ECM degradation and fibroblast activation, setting the stage for persistent tissue remodeling and fibrotic progression. The interplay between endothelial cells and CFs during this phase reinforces cytokine production, which may exacerbate myocardial inflammation, promote myofibroblast differentiation, and prime the heart for long-term structural remodeling.

### 5.6. Early Onset Chronic Phase

This transitional phase represents the evolution of acute injury into chronic myocardial damage [12]. It marks a range from the initial injury to the sustained structural remodeling. During this period, sustained cardiac stressors prompt CFs to undergo differentiation into α-SMA-positive myofibroblasts, reinforcing a self-amplifying fibrotic loop [139]. DOX-exposed CFs exhibit increased secretion of TGF-β, driving SMAD2 phosphorylation and sustained myofibroblast activation [22,67,139] (Table 2). Two weeks following DOX administration, isolated CFs retain this activated phenotype, with upregulated CTGF, galectin-3, MMP-2/9, and α-SMA in tissue lysates, histology and immunoblotting, confirming ongoing matrix deposition [67]. Concomitant increases in fibronectin and focal adhesion proteins further strengthen cell–matrix anchoring typical of the activated state [132]. DOX also elevates MMP-1 expression and activity in HCFs via PI3K/Akt signaling—an effect not seen in dermal fibroblasts suggesting simultaneous degradative remodeling that may weaken the ECM scaffold [22,94]. Together, these converging molecular and cellular mechanisms, including suppressed ECM homeostasis, enhanced proteolytic activity, persistent fibroblast activation, and amplified inflammation, define the early chronic ECM injury landscape. This stage lays the groundwork for irreversible myocardial remodeling if left unchecked, highlighting the need for timely intervention to mitigate long-term AIC.

### 5.7. Late Onset Chronic Phase

Chronic anthracycline cardiotoxicity manifesting weeks in animals [108,114,140] to years in humans [141] after treatment is characterized by sustained ECM remodeling, progressive interstitial fibrosis, and declining ventricular function. Over time, CFs adopt a stable myofibroblast phenotype, continuously secreting type I and III collagens, fibronectin and periostin leading to matrix accumulation, increased myocardial stiffness, and impaired diastolic relaxation [67,98,107,134]. This persistent fibrogenic state is maintained by chronically upregulated intracellular pathways in CFs such as PI3K/Akt, MAPK, and TGF-β/Smad signaling remain activated, promoting myofibroblast survival and ECM gene transcription. CFs isolated from chronically DOX-treated hearts exhibit elevated α-SMA and collagen expression, sustained by autocrine TGF-β release and SMAD2/3 phosphorylation [22,100,142]. Oxidative stress via NADPH oxidases is central to chronic remodeling [39,143,144]. NOX2 expression stays high in DOX-treated CFs, driving persistent ROS production, pathological cross-linking of collagen fibers, and heightened fibrosis [144]. Genetic deletion or pharmacologic inhibition of NOX2 or NOX4 in animal models alleviates ECM deposition and preserves cardiac function, underscoring their key role in chronic anthracycline injury [143,144].

MMP dynamics shift during chronic injury; although initial MMP upregulation facilitates acute ECM turnover, chronic DOX exposure leads to sustained MMP-9 overexpression and activity, driving maladaptive remodeling and interstitial fibrosis [114]. In rodent models, plasma MMP-2/9 activities rise by four weeks post-DOX, with left ventricular MMP-2 activation by eight weeks changes that correlate with Akt activation, superoxide dismutase inhibition, and caspase-3 cleavage [114]. Chronic DOX also impairs cellular quality control; autophagy and mitophagy are suppressed, leading to mitochondrial dysfunction, ATP deficits, and further ROS generation that exacerbate ECM deposition [48,118] (Table 2). In a chronic rabbit model of daunorubicin-induced cardiomyopathy, proteomic analyses have revealed significant alterations in basement membrane proteins, including collagen types I and III, and extracellular matrix constituents. These molecular changes align with increased interstitial fibrosis and MMP-2 upregulation, reinforcing the progression of adverse cardiac remodeling [108]. Ultimately, these chronic molecular and structural insults result in an irreversible ECM signature, typified by excessive collagen cross-linking, loss of myocardial compliance, and functional deterioration. This maladaptive remodeling process drives the transition from subclinical myocardial dysfunction to overt heart failure in long-term anthracycline recipients.

## 6. Therapy Development and Future Directions

**Figure 2:**
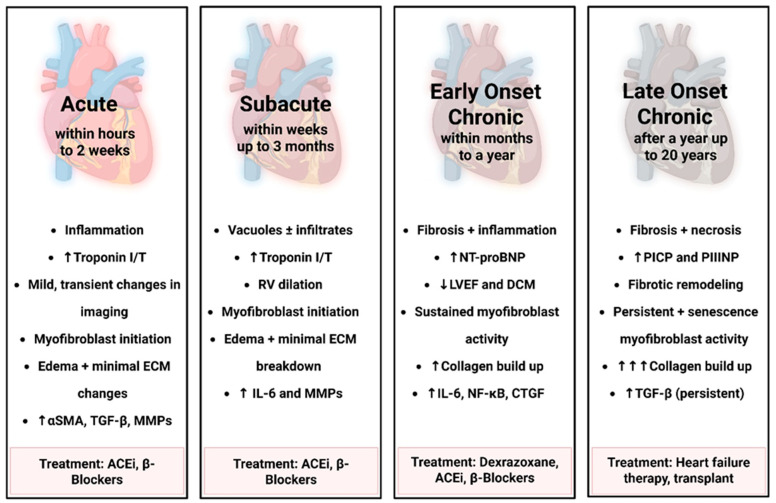
Temporal progression of Doxorubicin-induced fibroblast activation and ECM remodeling. This figure outlines the time-dependent stages of cardiac remodeling and fibroblast activation following doxorubicin exposure, ranging from acute to late-onset chronic based on human timeline. Early stages involve inflammation, edema, and myofibroblast initiation, while chronic stages are marked by sustained fibroblast activity, progressive fibrosis, and ECM remodeling. Biomarker elevation and functional decline reflect worsening cardiac injury. Corresponding treatments across stages include ACE inhibitors, β-blockers, Dexrazoxane, and advanced heart failure therapies. “Created in BioRender. Ibrahim, S. (2025) https://BioRender.com/iw02hrv”.

Both acute and chronic AIC can follow either reversible or irreversible courses, dependent upon the severity of cellular injury and the promptness of therapeutic intervention (Figure 2). The potential for recovery appears to be closely tied to early detection and prompt intervention [11,51]. Close monitoring and early intervention of LVEF post-chemotherapy show promising results in treating or lessening the burden of AIC. However, chronic or prolonged symptoms may be less responsive, reducing the recovery rate compared to acute cases. Despite this variability, management of DOX-induced cardiomyopathy remains predominantly supportive, relying on standard heart failure regimens. Treatment of anthracycline-induced cardiotoxicity can be broadly categorized into two critical therapeutic windows: (1) prophylactic or preventive interventions administered concurrently with anthracyclines particularly in high-risk patients [4,12] and (2) early therapeutic interventions initiated during the early chronic phase, characterized by a decline in LVEF without overt heart failure symptoms [12].

Conservative management remains the cornerstone of care for patients receiving anthracyclines, including regular cardiac imaging, control of cardiovascular risk factors, and biomarker surveillance [12]. Early detection of subclinical left ventricular dysfunction such as declines in LVEF or abnormalities in myocardial strain enables the timely initiation of guideline-directed medical therapy [9,11,12]. Conventional heart failure treatments, including angiotensin-converting enzyme (ACE) inhibitors and β-blockers, have been shown to halt or even reverse the progression to symptomatic heart failure when introduced early [11,145,146]. Among β-blockers, carvedilol offers an added benefit due to its combined β-adrenergic blockade and antioxidant properties. Clinical trials report that initiating carvedilol during or soon after anthracycline exposure can preserve LVEF, improve diastolic function, and attenuate biomarker elevations such as troponin I and NT-proBNP [146,147]

In addition to pharmacologic heart failure therapies, a range of non-specific cardioprotective strategies have been evaluated especially during the early subclinical or chronic phases of AIC. Antioxidants such as Coenzyme Q10, have shown promise in preclinical models by reducing ROS generated during anthracycline metabolism [148]. However, clinical evidence supporting their efficacy remains limited and inconclusive [9,11,12].

AIC remains inevitable in a significant subset of patients, even with modern cardioprotective regimens [9,17]. While evolving strategies now emphasize dose limitation, intensive cardiac surveillance, the development of targeted drug delivery systems may also minimize off-target toxicity. One of the most promising approaches is nanotechnology-based drug formulation. For example, pegylated liposomal doxorubicin (PLD), commercially known as Doxil, encapsulates doxorubicin within liposomes coated with polyethylene glycol [149,150]. This formulation prolongs systemic circulation, improves pharmacokinetic stability, and restricts tissue distribution primarily to tumor sites via enhanced permeability and retention effects [150]. PLD significantly reduces DOX-associated cardiotoxicity and other systemic side effects without compromising antitumor efficacy [149].

Given the central role of ECM integrity in maintaining myocardial structure and function, intervening in matrix-degrading pathways particularly MMPs and their tissue inhibitors (TIMPs) emerges as a promising strategy for both biomarker development and therapeutic mitigation of adverse remodeling [76,77,124]. DOX-induced upregulation of MMP1 in HCFs is mediated via PI3K/Akt signaling, and pharmacologic inhibition of PI3K/Akt abolishes MMP1 production, thereby preserving ECM homeostasis and potentially forestalling the progression to cardiac dysfunction and fibrosis [22,73]. A study about the effect of DOX on ECM has shown that a threefold increase in collagen deposition following DOX returns to baseline levels upon MMP inhibition [73]. Oral administration of MMP inhibitors such as doxycycline or ONO-4817 effectively prevents DOX-induced systolic and diastolic impairment, restoring fractional shortening and ejection fraction toward control values by stabilizing the interstitial ECM and preventing adverse remodeling [73,77].

Studies have shown that substance P, a neuropeptide in the heart, promotes the development of fibrosis through the neurokinin-1 receptor (NK-1R) which is expressed in cardiomyocytes, and CFs [151,152]. A recent study suggested that blocking NK-1R can partially reduce cardiac fibrosis particularly in DOX-induced cardiotoxicity [153].

Higenamine, a plant-derived β2-adrenergic receptor agonist with cardiotonic and anti-apoptotic properties, may offer protective effects against anthracycline-induced cardiotoxicity [154,155]. In murine models, higenamine significantly attenuates doxorubicin-induced cardiac remodeling and cardiomyocyte apoptosis, primarily through the suppression of aberrant AMP-activated protein kinase (AMPK) activation; a stress response pathway implicated in mitochondrial dysfunction and cell death during anthracycline exposure [155]. It also attenuates CFs-induced fibrosis via inhibition of TGF-β1/Smad signaling [155,156].

Dexrazoxane is an FDA-approved cardioprotective agent indicated for the prevention of anthracycline-induced cardiotoxicity in selected high-risk populations, including pediatric patients and adults with metastatic breast cancer receiving high cumulative doses of doxorubicin [157]. Its primary mechanism involves intracellular iron chelation, thereby limiting iron-catalyzed formation of ROS, which are key drivers of oxidative myocardial injury in anthracycline-treated patients [158,159,160,161]. In addition to its antioxidant properties, dexrazoxane is a strong catalytic inhibitor of Top2β in cardiomyocytes, preventing DNA double-strand breaks and mitochondrial dysfunction central mechanisms in anthracycline-induced cardiac damage [10,162]. Clinical trial studies have consistently demonstrated that dexrazoxane reduces the incidence of cardiac events and preserves LVEF without compromising the antitumor efficacy of DOX [63].

DOX exposure markedly increases the mRNA expression of pro-inflammatory cytokines such as tumor necrosis factor-α (TNF-α) and interleukin-6 (IL-6) [137]. An experimental study including pretreatment with fibroblast growth factor 21 (FGF21) significantly suppresses this cytokine upregulation, highlighting its potential as a targeted modulator of inflammation in AIC. This cardioprotective effect is mediated through its potent anti-inflammatory, antioxidant, and anti-apoptotic activities. FGF21 downregulates pro-inflammatory cytokines such as TNF-α and IL-6 by inhibiting the IKK/IκBα/NF-κB signaling pathway. Its antioxidant action involves reducing the generation of ROS, partly through modulation of nuclear factor erythroid 2–related factor 2 (Nrf2), a key regulator of cellular redox homeostasis. Additionally, FGF21 exerts anti-apoptotic effects by suppressing apoptotic signaling, thereby improving cardiac cell survival [137]. Collectively, these mechanisms contribute to the overall reduction in myocardial inflammation, oxidative stress, and apoptosis following anthracycline exposure.

Recent preclinical studies have suggested that mast cell-mediated inflammation may contribute to the progression of cardiac injury during the vulnerable window between initial myocardial insult and overt heart failure [163,164]. Mast cell stabilization may play a protective role in subacute anthracycline-induced cardiotoxicity. Cromolyn sodium, a mast cell stabilizer and histamine release inhibitor traditionally used to treat allergic conditions, has shown potential in reducing doxorubicin-associated myocardial inflammation and remodeling [15,165]. In rodent models, cromolyn administration attenuated mast cell degranulation, decreased myocardial histamine levels, and improved left ventricular function, particularly when administered during the early subclinical phase following DOX exposure [15].

Moving forward, identifying temporally regulated molecular signatures of ECM remodeling in anthracycline-induced cardiotoxicity is critical for developing targeted, phase-specific therapies aimed at preventing long-term cardiac dysfunction in cancer survivors. Advanced cardiovascular imaging plays a central role in pre-chemotherapy risk stratification and in detecting fibroblast-driven myocardial changes across acute, subacute, and chronic phases of AIC. Early detection of subclinical dysfunction using echocardiography and circulating biomarkers, followed by prompt treatment, can prevent progression to LVEF decline, supporting the potential reversibility of early myocardial injury. Thus, integrating imaging and biomarker data into predictive computational models may further enhance cardiotoxicity risk assessment and support the development of personalized prevention strategies.

## Figures and Tables

**Table 1:** Anthracycline molecular mechanism.

Mechanism	Primary Effect	Relevance to ECM Remodeling
ROS/redox cycling	mtDNA damage, lipid peroxidation	Early fibroblast stress and cytokine release
Topo IIβ inhibition	DNA double-strand breaks	Senescence and cell-cycle arrest in CFs
Mitochondrial dysfunction	ATP depletion	Impaired matrix turnover, apoptosis
SASP induction	IL-6, TGF-β secretion	Paracrine signaling → myofibroblast activation

**Table 2:** Time-dependent effects of doxorubicin on ECM remodeling and pathways.

Pathway	Acute	Subacute	Early Chronic	Late Chronic
Oxidative stress	↑ROS and↑NOX2/4 mtDNA damage	Persistent but lower ROS; redox-sensitive gene programs	Mitochondrial dysfunction maintains low-grade ROS	ROS–fibrosis feed-forward w/stiffness
TGF-β/SMAD	Transient surge from injury	Sustained activation; myofibroblast priming	Myofibroblast stabilization; ↑ECM synthesis	Pro-fibrotic set-point; ↑ cross-linking
MMP/TIMP	MMP-2/9 activation → early proteolysis	Remodeling window; ECM turnover	Shift to TIMP-biased balance	Net accumulation, ↑collagen I/III

## Data Availability

No new data were created or analyzed in this study as it is a review manuscript.

## Abbreviations

The following abbreviations are used in this manuscript:

α-SMA	α-Smooth Muscle Actin
ATP	Adenosine Triphosphate
PIIINP	Amino-Terminal Propeptide of Procollagen Type III
AMPK	AMP-Activated Protein Kinase
ACE	Angiotensin-Converting Enzyme
AIC	Anthracycline-Induced Cardiotoxicity
BNP	B-Type Natriuretic Peptide
PICP	Carboxy-Terminal Propeptide of Procollagen Type I
CCT	Cardiac Computed Tomography
CFs	Cardiac Fibroblasts
cTnI	Cardiac Troponin I
cTnT	Cardiac Troponin T
CMR	Cardiovascular Magnetic Resonance
CCN2	Cellular Communication Network Factor 2
CTGF	Connective Tissue Growth Factor
DNA	Deoxyribonucleic Acid
DOX	Doxorubicin
ECM	Extracellular Matrix
ECV	Extracellular Volume
FGF21	Fibroblast Growth Factor 21
GLS	Global Longitudinal Strain
GDF-15	Growth Differentiation Factor-15
HCFs	Human Cardiac Fibroblasts
IKK	IκB kinase
IκBα	Inhibitor of IκBα
IL	Interleukin
LV	Left Ventricular
LVEF	Left Ventricular Ejection Fraction
MMP	Matrix Metalloproteinase
MPO	Myeloperoxidase
NT-proBNP	N-terminal fragment of BNP
NTT-MMP	N-Terminal Truncated MMP
NK-1R	Neurokinin-1 Receptor
NHDFs	Normal Human Dermal Fibroblasts
NFκB	Nuclear Factor κB
Nrf2	Nuclear Factor Erythroid 2-Related Factor 2
PLD	Pegylated Liposomal Doxorubicin
PI3	Phosphoinositide 3-kinase
AKT	Protein kinase B
ROS	Reactive Oxygen Species
RNA	Ribonucleic Acid
RV	Right Ventricle
SASP	Senescence-Associated Secretory Phenotype
sST2	Soluble Suppression of Tumorigenicity-2
STE	Speckle-Tracking Echocardiography
TIMP	Tissue Inhibitor of Metalloproteinases
Top2β	Topoisomerase IIβ
TGF	Transforming Growth Factor
TNF	Tumor necrotic factor
